# Effects of Antidepressants on COVID-19 Outcomes: Retrospective Study on Large-Scale Electronic Health Record Data

**DOI:** 10.2196/39455

**Published:** 2023-04-11

**Authors:** Md Mahmudur Rahman, Atqiya Munawara Mahi, Rachel Melamed, Mohammad Arif Ul Alam

**Affiliations:** 1 The Richard A Miner School of Computer & Information Sciences University of Massachusetts Lowell, MA United States; 2 Department of Biological Sciences University of Massachusetts Lowell Lowell, MA United States; 3 Department of Medicine University of Massachusetts Chan Medical School Worcester, MA United States

**Keywords:** causal inference, treatment effect, drug effect, COVID-19 outcomes, COVID-19 severity, drug repurposing, COVID-19, depression, mental health, data mining, electronic health record, machine learning, antidepressant, causal inference method

## Abstract

**Background:**

Antidepressants exert an anticholinergic effect in varying degrees, and various classes of antidepressants can produce a different effect on immune function. While the early use of antidepressants has a notional effect on COVID-19 outcomes, the relationship between the risk of COVID-19 severity and the use of antidepressants has not been properly investigated previously owing to the high costs involved with clinical trials. Large-scale observational data and recent advancements in statistical analysis provide ample opportunity to virtualize a clinical trial to discover the detrimental effects of the early use of antidepressants.

**Objective:**

We primarily aimed to investigate electronic health records for causal effect estimation and use the data for discovering the causal effects of early antidepressant use on COVID-19 outcomes. As a secondary aim, we developed methods for validating our causal effect estimation pipeline.

**Methods:**

We used the National COVID Cohort Collaborative (N3C), a database aggregating health history for over 12 million people in the United States, including over 5 million with a positive COVID-19 test. We selected 241,952 COVID-19–positive patients (age >13 years) with at least 1 year of medical history. The study included a 18,584-dimensional covariate vector for each person and 16 different antidepressants. We used propensity score weighting based on the logistic regression method to estimate causal effects on the entire data. Then, we used the Node2Vec embedding method to encode SNOMED-CT (Systematized Nomenclature of Medicine-Clinical Terms) medical codes and applied random forest regression to estimate causal effects. We used both methods to estimate causal effects of antidepressants on COVID-19 outcomes. We also selected few negatively effective conditions for COVID-19 outcomes and estimated their effects using our proposed methods to validate their efficacy.

**Results:**

The average treatment effect (ATE) of using any one of the antidepressants was −0.076 (95% CI −0.082 to −0.069; *P*<.001) with the propensity score weighting method. For the method using SNOMED-CT medical embedding, the ATE of using any one of the antidepressants was −0.423 (95% CI −0.382 to −0.463; *P*<.001).

**Conclusions:**

We applied multiple causal inference methods with novel application of health embeddings to investigate the effects of antidepressants on COVID-19 outcomes. Additionally, we proposed a novel drug effect analysis–based evaluation technique to justify the efficacy of the proposed method. This study offers causal inference methods on large-scale electronic health record data to discover the effects of common antidepressants on COVID-19 hospitalization or a worse outcome. We found that common antidepressants may increase the risk of COVID-19 complications and uncovered a pattern where certain antidepressants were associated with a lower risk of hospitalization. While discovering the detrimental effects of these drugs on outcomes could guide preventive care, identification of beneficial effects would allow us to propose drug repurposing for COVID-19 treatment.

## Introduction

The COVID-19 outbreak [1], which was declared a pandemic in 2020 [2], is a devastating health crisis that needs new preventive strategies and treatments. One characteristic distinguishing this pandemic from others is the remarkable heterogeneity of outcomes among infected people. While some patients have mild illness, 18% have moderate or severe outcomes [3,4]. Worse outcomes have been associated with several risk factors, including age [5], sex [6-8], socioeconomic background, and comorbidities, such as obesity [9-11], chronic obstructive pulmonary disease [12-15], type 2 diabetes [16,17], and hypertension [18-20]. Yet, these risk factors do not fully explain the variation in outcomes. Some drugs may change the course of COVID-19 [21,22]. Discovering these either beneficial or harmful effects could improve medical care. For instance, certain cyclooxygenase inhibitors, which are common anti-inflammatory drugs, have been associated with worse outcomes, suggesting that some pain relievers should be avoided in COVID-19 patients [23]. On the other hand, discovering medications associated with improved outcomes can help us identify new therapies. From the early stages of the outbreak, a number of drugs have been proposed for repurposing, including hydroxychloroquine, which was notorious, and remdesivir, a broad-spectrum antiviral, which was successful [24]. Because the SARS-CoV-2 virus targets the renin-angiotensin-aldosterone system through its interaction with the ACE2 receptor, previous investigations have used the cohort study method to investigate infections and outcomes in people taking ACE inhibitors or angiotensin receptor blockers [25]. Those results indicated that a protective effect could be identified from retrospective analyses of people on these medications.

Building on these encouraging findings, we aimed to discover whether other classes of medications could impact outcomes. We focused on antidepressants, which are common drugs used by over 13% of adults in the United States [26]. Antidepressants have been linked to unexpected effects on diverse inflammatory and cardiovascular outcomes [27]. Use of antidepressants has been associated with an increased risk of hospital mortality [28], possibly due to their cardiovascular effects [27]. In a small study in France, protective effects on severe COVID-19 outcomes were found [29]. In another study, Clelland et al [30] showed a significant protective association between antidepressant use and COVID-19. Hoertel et al [31] showed a protective effect of fluoxetine or fluvoxamine on COVID-19 mortality. In a separate study, Hoertel et al [29] used a Cox regression model to investigate the association between antidepressant intake and the risk of intubation and COVID-19 mortality. Because of the popularity of antidepressants and their previous associations with complications that are relevant to COVID-19 outcomes [32,33], we investigated the possible effects of antidepressants on COVID-19 using a large population in the United States.

While previous work has assessed the association of medication use with COVID-19 severity [34], including the studies mentioned above, the work was limited by both a small population size and minimal adjustment for confounding. For instance, Israel et al [34] estimated the effects of drugs on COVID-19 using a case-control method. They matched COVID-19 cases against a control cohort of COVID-19–negative people on a set of 5 to 12 selected confounders. This limited adjustment for confounding is typical of previous studies, which have not controlled for possible confounders, including history of high cholesterol and other recorded medical care. Factors, such as socioeconomic status, can influence disease risk and influence levels of medical care, and such factors could create confounding. It is possible that people with more medical care, including those diagnosed and in treatment for other medical conditions, are at reduced risk for complications. If all confounders are known and well measured, then such an approach could work well. However, given the lack of knowledge about the risk factors for COVID-19, approaches controlling for a minimal set of possible confounders are vulnerable to residual confounding. It is crucial to critically assess the methods used for estimating these results.

Here, we made use of the National COVID Cohort Collaborative (N3C), a database aggregating health history for over 12 million people in the United States, including over 5 million with a positive COVID-19 test. In addition to health history for each COVID-19 patient, this data set provides a severity score for each patient, based on the World Health Organization COVID-19 severity scale [3]. To estimate the effects of antidepressants on COVID-19 outcomes, we considered 16 antidepressants from the N3C data enclave, with each used by more than 5000 patients. To estimate the effects of these drugs on outcomes, we applied causal inference methods, including a novel application of health embeddings. Our methods build on approaches like the case-control or cohort study to estimate effects of exposures on an outcome of interest. While measurement of true causal effects requires a randomized trial, such approaches are expensive and unlikely to be performed for every common drug. Instead, causal inference methods aim to emulate a clinical trial using observational health data. While the resulting inferences cannot be conclusively deemed causal, they represent our best possible estimate using nonexperimental data. Therefore, unlike previous work, we used causal inference methods to rigorously adjust for confounding. We used both a well-established method (high-dimensional propensity score [35] weighting) and a relatively less common method based on embeddings of medical codes [36]. The contributions of this work include both the estimates of the effects of antidepressants, and the rigorous assessment and comparison of methods for causal effect inference.

## Methods

### Data Sources

Our analysis made use of the N3C resource, which aggregates data on over 12 million people in the United States across dozens of sites of care. This population includes over 5 million people with COVID-19. Data sources were united using the OMOP Common Data Model, which allowed common concept identifiers to be created and a common format to be achieved across diverse data sources. An application under the Data Use Request system allowed us to access the deidentified version of the data. These data create a comprehensive portrait of the health history of millions of people, with loss of only exact dates and exact locations for each person.

From the set of COVID-19–positive people, we obtained their subsequent severity score previously calculated based on the World Health Organization index [3]. For each person, this is the most severe encounter in their medical history, based on a 5-level scoring system. The levels are mild, mild with emergency department visit, moderate with hospitalization, severe with hospitalization, and hospital mortality. Because of the small size of the population with a severe condition or mortality, we grouped together all hospitalized patients (around 20% of the positive population) to identify how antidepressant use affects hospitalization. People missing a severity score were considered as nonhospitalized, since any record of hospitalization is likely to have been noted in the health record. Therefore, our focus was on identifying causal effects on the presence of the hospitalization outcome. Notably, this measure has been previously used to assess demographic factors associated with COVID-19 outcomes [3].

### Study Population

We identified 16 common antidepressants using the OMOP concept relation data. First, we obtained all concepts of the type “ingredient” that are descendants of the ATC class “antidepressants” (OMOP concept ID 21604686). Then, we obtained all drugs that contained these ingredients and obtained all instances of use of these drugs, using the condition_era table in N3C. We retained all ingredients used by more than 5000 people to create a set of 16 antidepressants.

Antidepressants are divided into 5 classes based on which neurotransmitter they affect. Among the 16 antidepressants we considered for our study, fluoxetine, paroxetine, sertraline, citalopram, and escitalopram are classified as selective serotonin reuptake inhibitors (SSRIs); duloxetine, venlafaxine, and desvenlafaxine are classified as serotonin and norepinephrine reuptake inhibitors (SNRIs); trazodone, mirtazapine, vortioxetine, vilazodone, and bupropion are classified as atypical antidepressants; and nortriptyline, amitriptyline, and doxepin are classified as tricyclic antidepressants [37]. Monoamine oxidase inhibitors are a type of antidepressant that can cause potentially serious side effects, and they are rarely prescribed by doctors nowadays [37]. Moreover, our data set had no data points that involved antidepressants from the monoamine oxidase inhibitor class. Hence, we ignored this class in our study.

**Figure 1:**
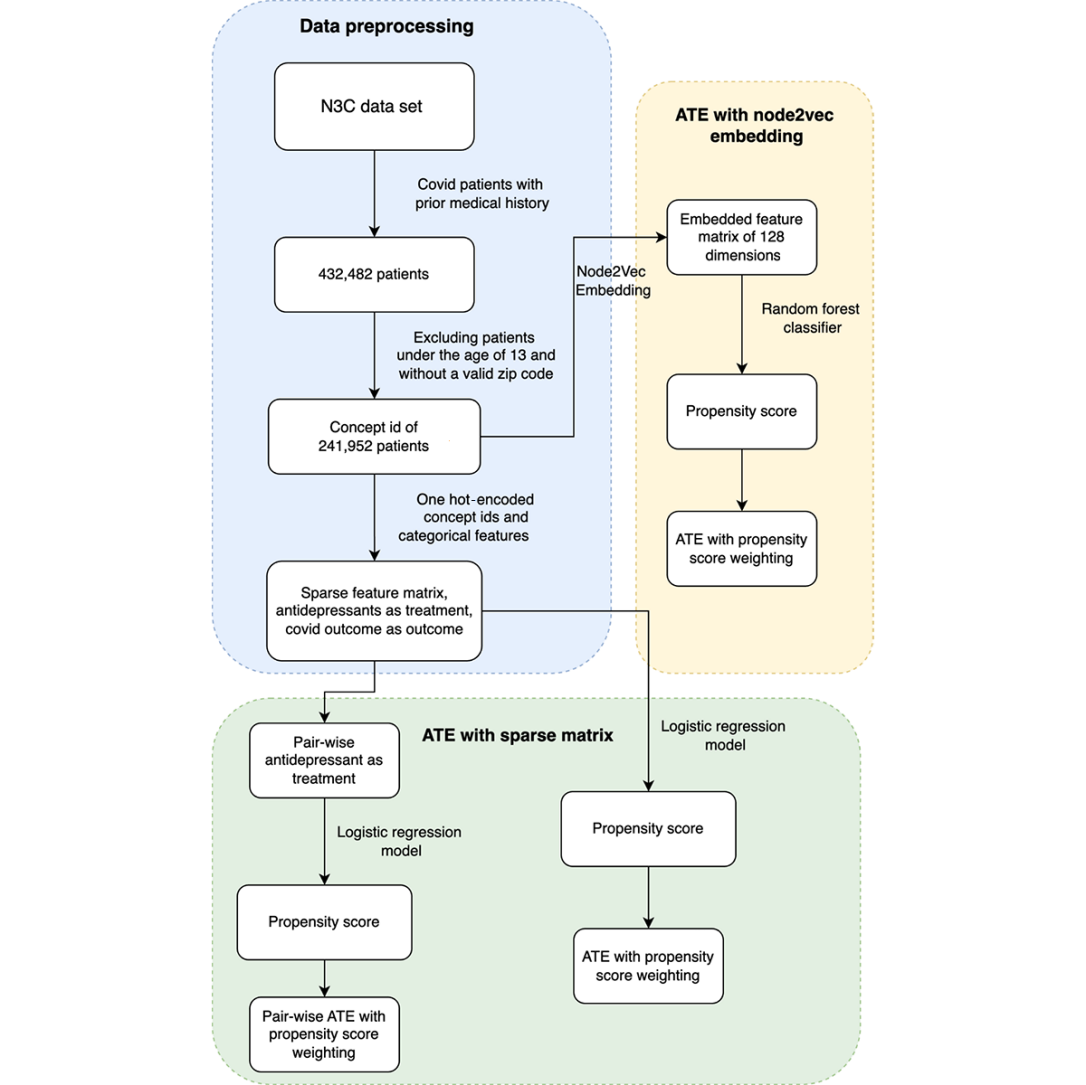
Overview of causal inference estimates and methods for obtaining them. ATE: average treatment effect; N3C: National COVID Cohort Collaborative.

Among the COVID-19–positive population, we further restricted our analysis to those who had a medical history of at least 1 year. This is common in pharmacoepidemiology studies to obtain an adequate history of the study population. We further restricted our study to those with an age of over 13 years and with a valid zip code. Eventually, we identified 241,952 individuals taking one or more antidepressants (Figure 1).

### Ethical Considerations

In this study, we used deidentified observational data from the N3C [38], and this is not considered human subject research. N3C has approved the data for secondary use without the need for institutional review board approval and has approved this study for publication/submission.

### Causal Inference Analysis

We performed multiple analyses to assess the causal effect while controlling for all measured confounders. First, we obtained all health history and demographics preceding the positive COVID-19 diagnosis for each patient in the data set. This set of variables consisted of all possible diagnosis codes (from the “condition_era” table), as well as age, gender, race, ethnicity, and the 3-digit zip code (from the “person” table).

We identified a number of treatment effects of interest. We were interested in estimating the effect of taking each antidepressant versus not taking each antidepressant (nonuser analysis), and additionally the difference in effects for each pair of antidepressants (active comparator analysis). Each treatment effect of interest identifies a pair of populations: the treated cohort (all COVID-19–positive people who are taking the drug of interest) and the comparison cohort (all COVID-19–positive people who are either not taking the drug of interest in the nonuser analysis or are taking another drug in the active comparator analysis).

The average treatment effect (ATE) was defined as the mean difference in outcomes between the two cohorts. If *h_i_* represents the hospitalization outcome for the *i*th person, the formula for the ATE is as follows:



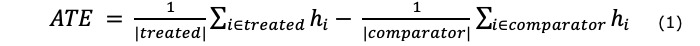



One major issue with the use of an observational data set instead of performing a randomized controlled trial is the risk of having selection bias in the experimental setup. We expect the pair of cohorts to differ in terms of health history and demographics, which can confound an unadjusted estimate of the ATE. Therefore, it is important to adjust for these differences to obtain an unbiased effect estimate. A common method to adjust for confounding is propensity score weighting, which creates a weighted pseudopopulation where treated comparator populations are balanced for possible confounders [39]. Lee et al [40] elaborately explained how machine learning models improve the performance of propensity score weighting. Pan et al [41] presented some references on how classification and regression models provide an improved version of propensity score weighting. Moreover, we included all medical history data of the patients as features, which resulted in high-dimensional input feature vectors (18,584-dimensional). Processing high-dimensional data is computationally expensive and is not feasible in almost all existing methods other than sparse logistic regression [42]. Considering this, here, we implemented propensity score weighting using sparse logistic regression and random forest. Both methods share the goal of representing possible confounders, and we performed 2 representations to avoid sensitivity to misspecification of the model for propensity. The propensity score represents the probability of each person falling into the treated or comparator cohorts, given their history and demographics, as follows:

p(treated | person i’s health history, demographics) = pi (2)

We estimated this propensity score using 2 different and complementary methods, and then, we used this score to weight each person’s overall contribution to the estimate of the ATE as follows:



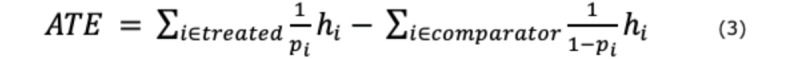



For each causal effect of interest, we estimated the propensity for treatment using 2 different ways of encoding health history (Figure 1). First, we estimated propensity for treatment by performing a high-dimensional regularized logistic regression, which was fitted to model *p_i_* separately for each causal effect of interest. For this analysis, we encoded each of the health history and demographic variables using the one-hot encoding scheme. Therefore, we modeled all previous diagnoses and treatments, creating a resulting 18,584-dimensional covariate vector for each person. Second, we used an embedding representation of patient health status at the time of the COVID-19–positive test. The embedding representation was precomputed by Pattisapu et al [36], using the Node2Vec method to encode SNOMED-CT (Systematized Nomenclature of Medicine-Clinical Terms) medical concepts to the embedded vector space. For each of the 18,584 health history codes, we matched the code to its 128-dimensional pretrained embedding vector *e_c_*. Then, for the *i*th person, given their list of previous medical codes *{codes_i_}*, we created an overall representation of patient health by averaging these vectors as follows:



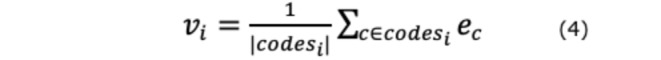



Then, we modeled the propensity for treatment given the vector of the patient health state. Logistic regression was not feasible for this large nonsparse data, so we used random forest, which is also a popular tool for estimating the propensity score. Here, the ATE was assessed on the full data set. We chose these methods because the first (high-dimensional propensity score) is the more standard method and the second (embedding) can potentially account for poorly measured confounders [43]. By performing both types of causal inferences, we can evaluate the sensitivity of our results to specifications of the propensity model. These approaches have the potential to adjust for confounding, unlike previous methods [29-31].

### Obtaining CIs

We obtained CIs using the bootstrap method. Specifically, we sampled with replacement to obtain our pair of cohorts. For each sample, we estimated the propensity weights and used this to estimate the overall causal effect. This process was repeated 100 times to create 100 estimates, providing the CIs.

### Assessing Our Results Using Negative Controls

The practice of using negative control outcomes, which are outcomes thought not to be causally affected by an exposure, is intended to form a point of comparison for our causal effects of interest. We selected negative control outcomes using literature on known causal effects of antidepressants, selecting some common outcomes that are not likely to be the result of antidepressant use. We selected the following: fracture of bone (SNOMED-CT code 125605004), asthma (SNOMED-CT code 195967001), chronic kidney disease (SNOMED-CT code 709044004), disorder of nail (SNOMED-CT code 17790008), and eczema (SNOMED-CT code 43116000). For each negative outcome, we estimated the causal effect in the same way as for our outcome of interest (hospitalization with COVID-19).

## Results

### Topics of Interest

Our main results addressed 2 topics of interest. First, we were interested in discovering new effects of drugs on COVID-19 outcomes, as measured by the severity score. Second, we wanted to evaluate our methods in order to contribute to the causal inference literature.

### Causal Effects of Interest

**Figure 2:**
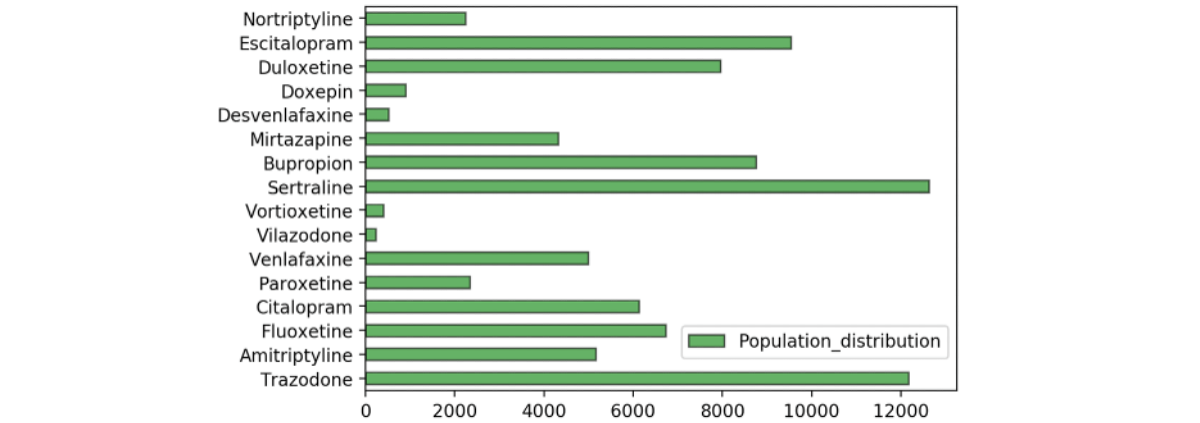
Population distribution for each antidepressant. This represents the number of patients who took each of the antidepressants as treatment.

In order to discover the actionable unknown effects of drugs on COVID-19 trajectory, we focused on a set of causal effects of interest. We were interested in the effect of each common antidepressant on COVID-19 hospitalization outcome. Figure 2 shows the frequency of prescription of each antidepressant in the population. We followed the approach of emulating randomized trials using observational data [44]. We created target randomized trials to follow the user versus nonuser design and to follow the active comparator design. In the user versus nonuser design, we emulated a trial where people are randomized to either using an antidepressant or not using an antidepressant. In the active comparator design, the target trial compared people taking one antidepressant versus another. Each such target trial defined 2 populations of interest: the treated and comparison populations. Then, for each causal effect of interest, we used multiple methods to estimate the relationship. Therefore, we performed one effect estimate for each antidepressant in a user versus nonuser design, and one estimate for each pair of antidepressants and comparison of each antidepressant to another in an active comparator design.

We obtained the population of people with a positive polymerase chain reaction test from the N3C data and obtained the score calculating the severity of their COVID-19 outcomes. We further identified those people with a history of taking antidepressants before their positive test. Using these data, we identified the treated and comparison cohorts for each effect of interest. To emulate a randomized trial, we must adjust for any medical history that may create a biased association between the treatment and outcome. We adjusted for all medical history data before the positive COVID-19 test using the propensity score weighting method to obtain the adjusted ATE (see Methods). We calculated CIs by creating 100 bootstrap samples of the data set (see Methods). We used 2 methods to encode medical history in order to calculate the propensity score: high-dimensional sparse representation of history, and representation by medical code embeddings. These 2 methods share the goal of representing possible confounders, but we intended for these 2 complementary representations to enable critical assessment of the methods and their effect estimates.

### Causal Effect Estimates Indicate a Significant Impact of Antidepressants on Hospitalization

**Figure 3:**
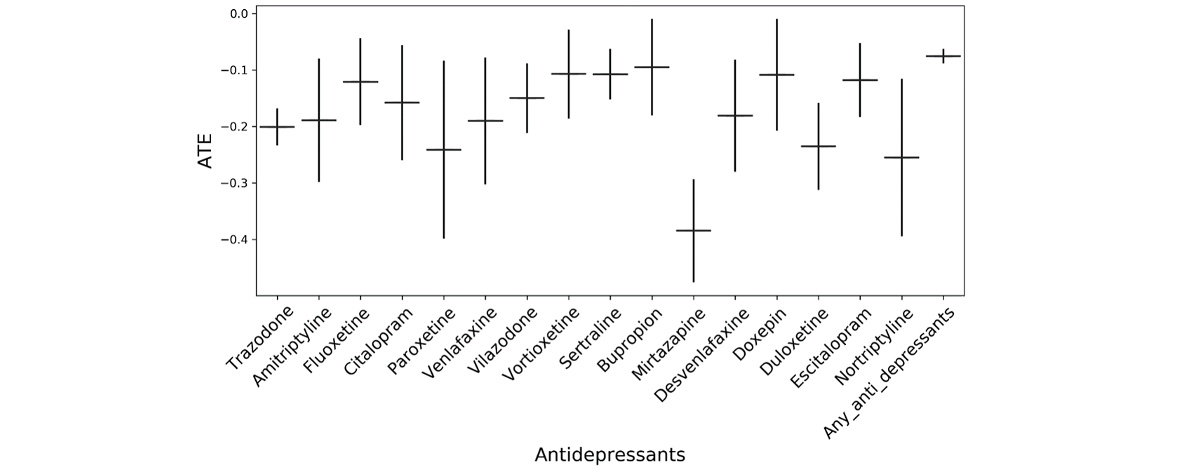
Average rate of nonhospitalization in the user versus nonuser design. ATE: average treatment effect.

**Figure 4:**
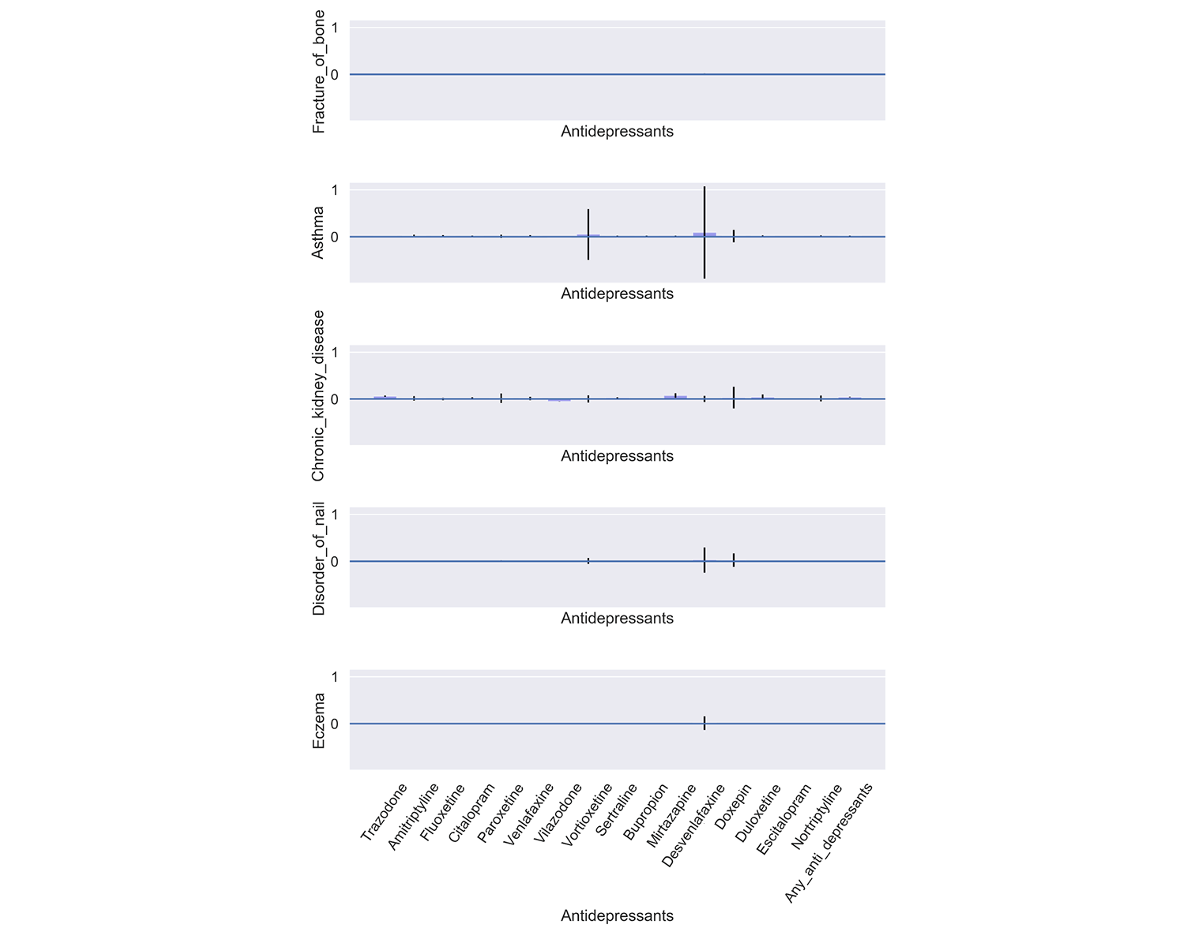
Negative control average treatment effects.

The results indicated a significantly worse outcome (higher rates of hospitalization among users) (Figure 3). In order to assess whether these results are specific to hospitalization outcomes or rather some difference in overall sickness between the 2 cohorts, we selected a set of negative control outcomes. Good negative controls are those that may be associated with confounding variables, such as overall sickness, but are not associated with the exposure of interest [45,46]. We used information on the known side effects of antidepressants to select 5 negative control outcomes (fracture, asthma, chronic kidney disease, nail disorder, and eczema) that were intended to represent diverse medical states unrelated to antidepressant use. While antidepressants showed significant associations with the outcome of interest (hospitalization), all negative control outcomes had no significant association with antidepressants (Figure 4).

### Active Comparator Design Suggests Differences in Outcomes Between Antidepressants

**Figure 5:**
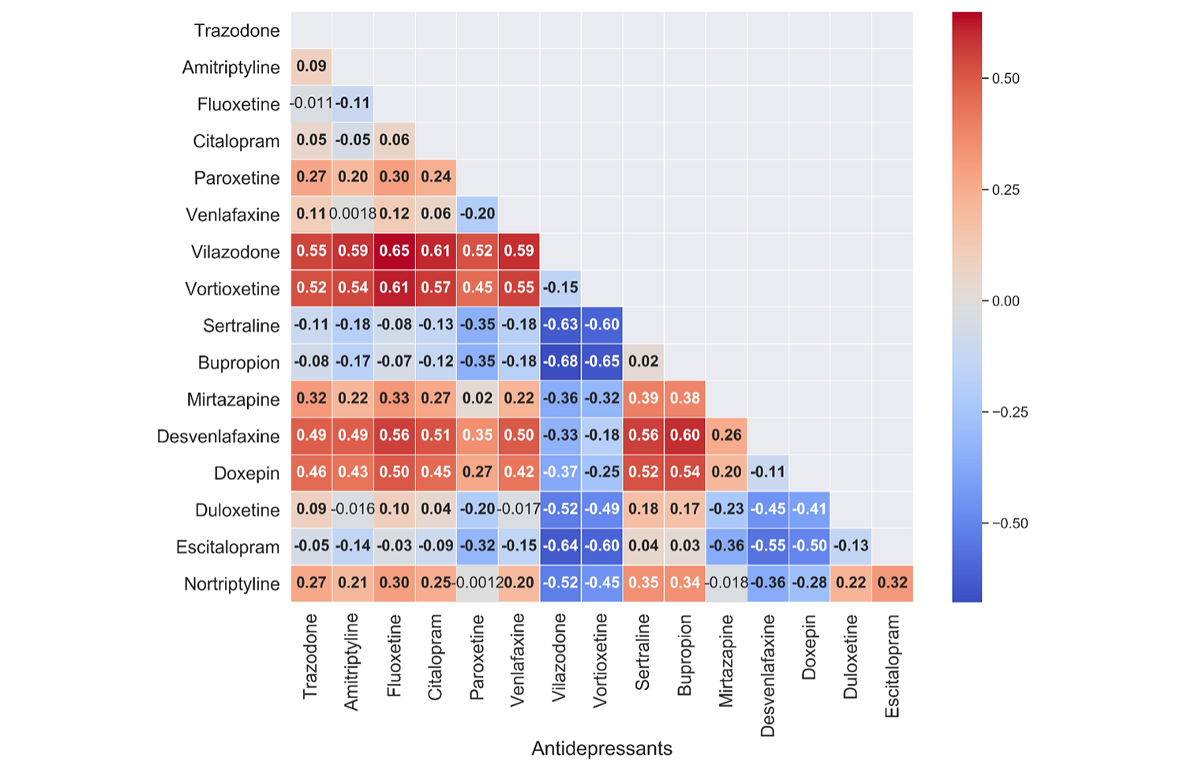
Results of the active comparator design comparing each antidepressant to others. The average treatment effect is shown in each box of the treatment (row) versus comparator (column), with bold numbers indicating a CI not overlapping the null effect.

While all antidepressants appeared to increase the risk of hospitalization, we also performed a head-to-head active comparator analysis to assess diversity in the effects. Vilazodone and vortioxetine, as compared to the other antidepressants, appeared to confer some protection against hospitalization. This may be due to uncharacterized cardiovascular effects, which have been described in certain contexts (Figure 5).

### Comparing the High-Dimensional Propensity Score Against the Embedding-Based Propensity Score

**Figure 6:**
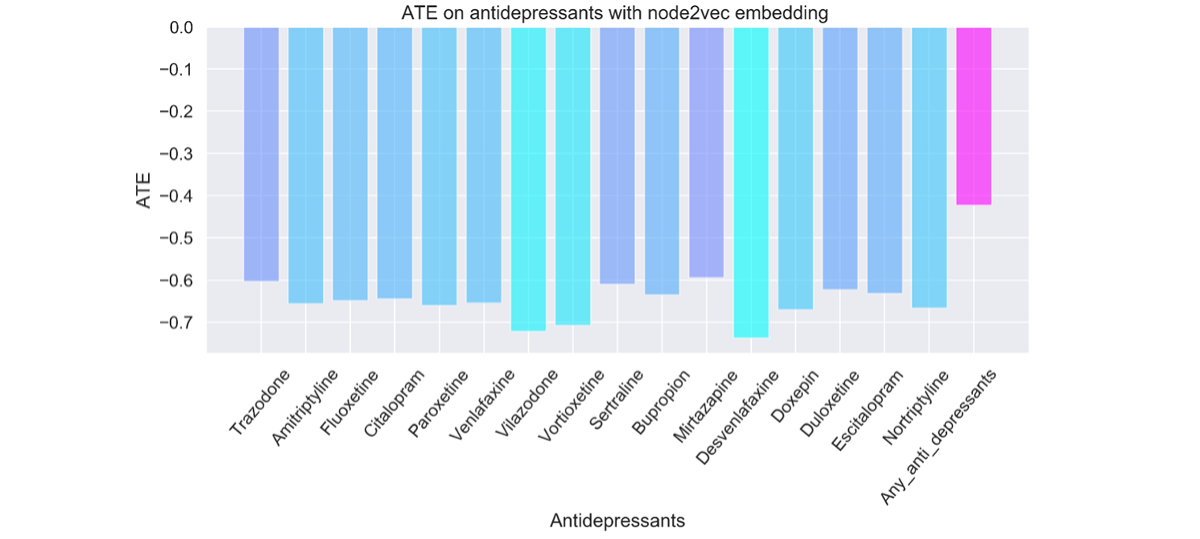
Average treatment effect using embedding representation of patient history. ATE: average treatment effect.

The high-dimensional propensity score required a sparse encoding that did not on its own capture any meaning of medical codes. That is, each of the 10,000 codes modeled in the propensity score must be modeled using a one-hot vector, and all context for each code is lost. This means that 2 medical codes for very similar conditions, for example, fracture of the right leg and fracture of the left leg, are encoded no more similarly than 2 medical codes for unrelated conditions. In order to examine the impact of a coding system that retains the meaning of the medical codes, we made use of medical embeddings that exist for the SNOMED-CT concept coding system [36]. While embeddings have been explored previously for matching [43], the use of embeddings to create propensity score weights has not been reported to our knowledge. We created an embedding representation of patient health history using the average of all medical codes in a person’s history. Then, as with the high-dimensional sparse representation of health history, we calculated propensity weights and obtained the ATE. The ATEs were much more extreme using this method (Figure 6), and the negative control outcomes similarly had a biased result (not shown). This contrasts with the high-dimensional propensity weighting method, where the negative control outcomes, as expected, retain a null effect.

## Discussion

### Principal Findings

This study aimed to apply causal inference methods to discover whether taking any common antidepressants is associated with poor COVID-19 outcomes, and to compare different methods for assessing these effects. Our findings suggest that common antidepressants may increase the risk of COVID-19 complications. Additionally, in our analysis of the effect of each common antidepressant as compared to that of other antidepressants, we uncovered a pattern where certain antidepressants were associated with a lower risk of hospitalization. We also experimented with multiple methods for encoding health history to uncover causal effects. Because health data involve thousands of medical codes, representing each of these codes using a sparse representation can create a very large regression problem for propensity weighting. In addition, this representation of medical history does not make use of knowledge about the meaning of these medical codes. In order to make use of this information, we alternatively encoded medical history using embedding vectors created using the Node2Vec method. These embeddings have been extensively evaluated as an efficient representation of medical knowledge [36]. We found that both methods had a consistent direction of effect, but the effect estimates were more extreme using embeddings rather than the sparse encoding.

Our findings about the effects of antidepressants, if replicated in other data sets, could suggest that providers should change their uses of antidepressants to improve COVID-19 outcomes among high-risk groups. Among prior studies, Lenze et al [47] performed a randomized controlled trial of 152 patients and showed a significantly reduced risk of worse clinical outcomes in patients with symptomatic COVID-19 who were administered fluoxetine than in those who were administered placebo. Oskotsky et al [48] performed an observational study on COVID-19 mortality and implemented a propensity score matching method only on the exposure of some specific SSRIs (fluoxetine or fluvoxamine). Moreover, several clinical and preclinical studies found an association between fluoxetine intake and a lower risk of intubation or mortality [29,49,50]. However, these findings do not answer if other SSRI or non-SSRI antidepressants are as effective as fluoxetine or fluvoxamine. In contrast, our study considered 16 antidepressants of 4 types, including SSRI, SNRI, atypical, and tricyclic antidepressants. The results indicated significant associations of citalopram, escitalopram, venlafaxine, desvenlafaxine, mirtazapine, doxepin, and vilazodone with a reduced risk of worse COVID-19 outcomes, apart from fluoxetine. Some other prior studies assessing the association between antidepressant intake and COVID-19 severity have used limited adjustment for confounders or small populations [29-31]. Our study has made extensive efforts to adjust for confounding. The results support and are consistent with the findings of prior preliminary studies. Further, our study showed that the association between antidepressant intake and a reduced risk of COVID-19 mortality, intubation, or a worse outcome is not only for fluoxetine or SSRIs, and other antidepressants from several classes have similar effects on COVID-19 outcomes. Our consistent results from 2 causal inference methods support that these methods can be used to mine possible effects from large health record data. As the methods are not specific to antidepressant advances, these findings suggest that the N3C data set could be used with these methods to investigate other factors impacting COVID-19 outcomes, including other drugs and other medical procedures and treatments.

### Limitations

In a nonrandomized setting, it is not possible to be certain that the results are free of residual confounding. Although our method carefully considered all medical history data, thus accounting for all measured confounding, unmeasured confounding could still bias the results. To mitigate this risk, we have undertaken an analysis using negative control outcomes. One possible example of unmeasured confounding is if people taking antidepressants generally have poor health. Poor health is not directly recorded in the N3C. However, in this case, we would expect an association between antidepressants and increased prevalence of many other diseases, such as the set of negative control outcomes we selected for analysis. Because we did not find any association of antidepressants with these negative control outcomes, our results do not appear to be due to this type of confounding. Other limitations include the short duration of observation for our data set, as we only used 3 years of data to estimate confounding. Some confounders may be recorded only more distantly in health history, but this time window is commonly used in observational data analysis. We also did not use the duration or dose of antidepressants; therefore, our results represent the impact of any use of antidepressants on disease outcomes.

Another caveat concerns the embedding effect estimates. This method estimated treatment effects that were much more extreme than those in the more traditional encoding of health history. Under a conservative interpretation, we believe this is more likely due to the shortcomings of this approach, which makes it susceptible to bias, rather than being due to a true extreme causal effect. The bias in the results could be due to one of the following reasons. First, the embedding vectors do not precisely represent important confounders. As the vectors are only 128-dimensional, some information about specific medical codes that may be crucial confounders may be lost. Second, the method for calculating propensity weights based on medical embeddings must be improved. This may involve developing other ways to represent a patient’s health history given a set of embeddings. Third, these embeddings are not designed to represent a patient’s state before drug prescription, and performance may be improved by applying medical embeddings specifically designed to represent the confounding relationship between health history and drug prescription [43]. Further experimentation is needed to assess how best to use embedding vectors for causal inference.

### Conclusions

In this study, we investigated how antidepressants affected COVID-19 outcomes, using causal inference methods. In addition to standard propensity score analysis, we implemented a novel application of health embeddings. To support the effectiveness of the suggested strategy, we also offered a novel drug effect analysis–based evaluation tool. This study used causal inference techniques on large electronic health record data to identify how commonly prescribed antidepressants affect hospitalization for COVID-19 or a worse outcome. The research suggested a pattern in which some antidepressants are connected to a decreased risk of hospitalization. Because the risk profile of antidepressants is well known, our findings can be used to provide justification for investment in future large-scale clinical trials to find the best treatment for depression in those with COVID-19 at high risk of poor outcomes. Future work can build on our methods to identify more factors influencing COVID-19 outcomes to help predict who is at high risk and to suggest interventions.

## Abbreviations

ATE: average treatment effect
N3C: National COVID Cohort Collaborative
SNOMED-CT: Systematized Nomenclature of Medicine-Clinical Terms
SNRI: serotonin and norepinephrine reuptake inhibitor
SSRI: selective serotonin reuptake inhibitor

## Data Availability

All the analyses were conducted using the National COVID Cohort Collaborative (N3C) database, which is available to researchers and investigators conducting COVID-19–related research, subject to certain eligibility criteria and data use agreements. Access to the data is granted through a secure online platform that requires registration and approval by the N3C Data Enclave Governance Committee. Researchers who are interested in using the cohort we generated from original N3C data, are asked to get N3C approval first and then contact the corresponding author, MAUA (mohammadariful_alam@uml.edu). For more information about accessing the N3C data and the eligibility criteria, please visit the N3C website [51].
